# Iss2Image: A Novel Signal-Encoding Technique for CNN-Based Human Activity Recognition

**DOI:** 10.3390/s18113910

**Published:** 2018-11-13

**Authors:** Taeho Hur, Jaehun Bang, Thien Huynh-The, Jongwon Lee, Jee-In Kim, Sungyoung Lee

**Affiliations:** 1 Department of Computer Science and Engineering, Kyung Hee University, (Global Campus), 1732, Deogyeong-daero, Giheung-gu, Yongin-si, Gyeonggi-do 17104, Korea; hth@oslab.khu.ac.kr (T.H.); jhb@oslab.khu.ac.kr (J.B.); thienht@oslab.khu.ac.kr (T.H.-T.); jwlee2oo@hanmail.net (J.L.); 2 Department of Smart ICT Convergence, Konkuk University, 120 Neungdong-ro, Gwangjin-gu, Seoul 05029, Korea

**Keywords:** human activity recognition, convolutional neural network, encoder, signal transformation, smartphone, smartwatch, accelerometer

## Abstract

The most significant barrier to success in human activity recognition is extracting and selecting the right features. In traditional methods, the features are chosen by humans, which requires the user to have expert knowledge or to do a large amount of empirical study. Newly developed deep learning technology can automatically extract and select features. Among the various deep learning methods, convolutional neural networks (CNNs) have the advantages of local dependency and scale invariance and are suitable for temporal data such as accelerometer (ACC) signals. In this paper, we propose an efficient human activity recognition method, namely Iss2Image (Inertial sensor signal to Image), a novel encoding technique for transforming an inertial sensor signal into an image with minimum distortion and a CNN model for image-based activity classification. Iss2Image converts real number values from the *X*, *Y*, and *Z* axes into three color channels to precisely infer correlations among successive sensor signal values in three different dimensions. We experimentally evaluated our method using several well-known datasets and our own dataset collected from a smartphone and smartwatch. The proposed method shows higher accuracy than other state-of-the-art approaches on the tested datasets.

## 1. Introduction

The purpose of human activity recognition (HAR) is to detect user behavior, such as locomotion, postures, and gestures, to understand users’ habits and lifestyles and provide healthcare and wellness services for health promotion. There are three different methods of HAR, video-based, wearable sensor–based, and environmental sensor–based, and each method has its own pros and cons.

In wearable sensor–based methods, devices containing inertial sensor units, such as an accelerometers (ACCs), gyroscopes and magnetometers, are attached to the body. Activities are then classified into types, with each activity type showing a different pattern of sensor values. To classify an activity, the features that can best represent it must be extracted from the collected sensory data. Selecting and extracting the most meaningful features are the most significant problems for achieving accurate HAR [1]. Traditionally, the features are chosen by humans, called hand-crafted features, and include time-domain features such as mean and standard deviation and frequency-domain features such as those found using the fast Fourier transform (FFT) [2]. To find the most efficient and effective features, (1) the programmers must have prior expert knowledge, or (2) they must do a large amount of empirical study to learn which features are useful [3]. Deep learning technology has now been developed and can be used for HAR [4]. The most prominent advantage of using deep learning is that it can automatically extract both low- and high-level features and select them without user manipulation. In terms of sensor signals, low-level features include statistical features and frequency-domain features, whereas high-level features are semantic [5]. Likewise, for images, low-level features are external representations of objects such as line or color, and high-level features are semantic [6]. In both cases, high-level features are understandable to humans. In the domain of HAR, low-level features can be signal lines, inflection points, min/max values, or variations, and high-level features are patterns in the signals.

Many researchers have used deep learning techniques for HAR, such as a convolutional neural network (CNN) [7], an autoencoder [8], a restricted Boltzmann machine [9], a recurrent neural network (RNN) [10], and a hybrid method [11]. Among those options, the CNN shows good performance in natural language processing, image recognition, and speech recognition [12]. CNNs are now widely used in inertial-sensor-based HAR and have two advantages over other techniques: local dependency and scale invariance [13]. Local dependency can show the correlation of successive values, and inertial data contain mainly temporal values. Scale invariance means that the intrinsic property is maintained, regardless of scale variability. For example, if a person walks for thirty minutes, their pace will vary over time without changing their activity, i.e., they are walking for the entire duration. Research using CNN for HAR usually is of two types, configuring the input data and configuring the CNN architecture [14]. Configuring the data involves changing the input inertial data into various different forms. For example, an ACC signal can be transformed into a raw plot image, with the *X*, *Y*, and *Z* axis signals projected into red, green, and blue channels, respectively, or they can be transformed into an image which shows frequency features such as spectrogram. Configuring the CNN architecture means to include new kind of layers or insert different layers from other deep learning techniques which does not belong to general CNN layers, such as other kinds of normalization or classification layers. From this perspective, our hypothesis is that transforming an inertial sensor signal into images with large dimensions will allow a CNN to infer many correlations among dimensions, allowing it to extract detailed features and rich information from the original signal.

In this paper, we use a data configuration method to propose an efficient HAR method, called Iss2Image. Iss2Image is a novel encoding technique for transforming an inertial sensor signal into an image-type data form because our CNN model uses image-based classification. The Iss2Image technique converts the real number values from the *X*, *Y*, and *Z* axes into three color channels to precisely infer correlations among successive sensor signal values in three different dimensions. Our method divides the sensor signal into three parts: the integer, the first two decimal places, and the following two decimal places. This technique minimizes data distortion without requiring any processes beyond dividing and extending the original signal. Other signal transformation methods, such as raw plot signal or spectrogram, are just drawing the signal in time series or the spectrogram of signal, and export to an image. These are not really an encoder where the signal value is not mapped/transformed to RGB pixel value. Besides, these methods depends on the resolution of the exported image. If the resolution is low, the signal is drawn dimly, not clear enough to see the signal, resulting downgrade of the accuracy. For our experiments, we used three well-known public datasets, compared with three pre-trained CNNs, and other traditional methods. In addition, we collected our own dataset using a smartphone and smartwatch, which are easily obtained and widely used. Many researchers are now adapting deep learning techniques for use with mobile device data [15,16,17,18,19], but previous works have focused on only a single device. We have experimentally compared the different datasets, different signal transformation methods, and different traditional methods with our method. The contributions of this paper are:

(1) A novel encoding technique for transforming an ACC signal into an image-type data form to precisely infer correlations among successive ACC signal values in three different dimensions.
(2) A fast and lightweight CNN-based activity recognition model for mobile platform.

The remainder of this paper is structured as follows: Section 2 describes related works for deep learning–based HAR; Section 3 describes our proposed methodology; Section 4 describes and discusses our experiments, and we present our final conclusions in Section 5.

## 2. Related Works

Many works have used deep neural networks with inertial sensor data for HAR. These works can be categorized as changing the form of sensory data, configuring the architecture, and comparing different features or classification methods.

Changing the form of sensory data is essential to achieving inertial sensor–based activity recognition using a CNN, which requires its input to be in image form. Garcia-Ceja et al. [20] has changed the sensor signal into recurrence plots, a distance matrices that capture temporal patterns in the signal, and classified with CNNs. Zhang et al. [21] used linear interpolation to get values of a sequence in specific proportional position to form new feature vectors to have a fixed dimensionality. And then they formed an image called data-band, a narrow matrix with a small row number and a large column number, which could grasp the position invariant. Jiang et al. [22] changed sensory data into image form by stacking the data row-by-row. Then, they applied a 2D discrete Fourier transform (2D DFT) and chose its magnitude as the activity image. They also applied a bi-class support vector machine (SVM) classifier to sort uncertain categories after CNN classification. Dehzangi et al. [23] used smoothed Wigner-Ville distribution (SWVD) as an input with early and late fusion after and before CNN to improve the performance based on multiple sensor devices. Alsheikh et al. [24] and Ravi et al. [25] changed ACC signals into spectrograms, a three-dimensional representation of changes in the acceleration energy content of a signal as a function of frequency and time. Aforementioned four papers commonly use time-frequency representation for image. 2D DFT represents the sensor signal in discrete plane image. Because it does not contain temporal information, it is unsuitable to show the continuity of the activity. Both SWVD and spectrogram are bilinear representations, where spectrogram is represented after squared Short-Time Fourier Transform and SWVD is represented applying 2D low-pass filter on WVD to remove cross-term affection [26]. While spectrogram has lower complexity with faster computation time than SWVD, it has lower resolution. These works transformed sensory data into images, but the original values could be distorted by the additional computations they required.

Numerous methods can be used to change the general CNN architecture to increase its performance. Ha et al. [27] did not changed the general CNN architecture, but used 2D kernels in both the convolutional and pooling layers to capture local dependencies over time and spatial dependencies across sensors. Yang et al. [28] placed a unification layer between the convolutional and fully connected layers, which unified the feature map output. Instead of simply concatenating the feature maps, they were trying to achieve parametric concatenation. Baldominos et al. [29] applied neuroevolution, which changes the CNN topology for better accuracy. Ordóñez et al. [30] used a hybrid model, combining a CNN and long short term memory (LSTM). Cho et al. [31] proposed a divide-and-conquer-based method using two stage learning. They first classified the activity based on static and dynamic characteristics and then classified the detailed activity. Using such two-stage learning sharpens the data. Those works showed better results than using the general CNN architecture, but they also increased the computational complexity. Because those authors do not describe how they transformed the raw sensory data into an image, we cannot judge whether those methods always show better results than using a general CNN or another signal transformation method.

To find the best performance when deep learning techniques are used for sensor-based HAR, comparison studies have been conducted with different criteria. Hammerla et al. [32] compared deep feed forward networks, CNNs, and RNNs. Murad et al. [33] compared unidirectional, bidirectional, and cascaded architectures using an LSTM-based RNN that could capture long-range dependencies in variable-length input sequences. Saez et al. [34] compared classification methods, such as distance-based, statistical, kernel, decision tree, ensemble, and deep learning methods. Li et al. [35] compared feature learning from different methods: hand-crafted, multi-layer-perceptron (MLP), CNN, LSTM, hybrid CNN and LSTM models, an autoencoder, and codebook feature-learning approaches. Among those options, the hybrid model showed the best accuracy. To achieve minimum computational complexity with the highest performance, we transform the sensory data in an image form and design fast and lightweight CNN-based activity recognition model to process it.

## 3. HAR Using a CNN with a Transformed Inertial Sensor Signal

**Figure 1:**
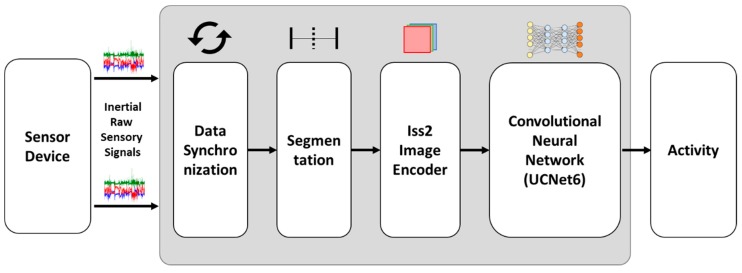
The workflow of our proposed method for human activity recognition; the main contributions are the data encoder and CNN model.

In this section, we explain our proposed method for transforming the inertial sensor signal using an encoding process and our CNN-based activity recognition learning procedure. First, we collected inertial raw sensory signals from a smartphone and smartwatch. The data from two different devices are synchronized to match each other and then segmented into 150 samples per 3 s window. Then, we transformed those data into color images using our proposed encoding method. The transformed signals were used as training input for a CNN, and then generated a CNN-based activity recognition model. Through this model, inertial raw sensory signal collected from a smartphone and smartwatch are input directly for classification, which produces the final activity label. Figure 1 shows the overall workflow.

### 3.1. Encoding the Inertial Sensor Signal

In this subsection, we will show the method using the ACC signal for example. The input to CNNs must be in an image form, a gray-scale or color image, with the pixel values represented as integers from 0 to 255. However, an ACC signal value is a real number containing both integers and decimal places, so it cannot be directly input into a CNN. Therefore, we here propose a novel encoder to efficiently transform raw ACC signal data into image data. We divide each ACC signal value (a real number) into three parts: the integer, the first two decimal places, and the next two decimal places. The three parts are separated and represented using decimal digits that correspond to the three color channels (red, green, and blue) of a color image. In that way, our proposed technique extends the dimensions of the original 1D ACC signal data into 3D image-type data. Every ACC signal value is modified, with the output represented as integers. For example, if one second of an ACC signal contains 150 values (50 *X* signal values, 50 *Y* signal values, and 50 *Z* signal values), each second of the encoded ACC signal contains a total of 450 integer values. If there are fewer than four decimal places, the missing places are set to zero. On the contrary, the ACC signal value will be rounded up to four decimal places.

Compared with the existing encoder, which uses only the integer to convert the ACC signal into a gray-scale image, our proposed technique more efficiently and precisely transforms the signal, minimizing distortion in the new image. Additionally, our transformation produces more detailed correlations among successive ACC signals than the earlier methods, not only among *X*, *Y*, and *Z* data, but also between their integers and decimal places. Given that one activity sample D, includes N samples of [*x y z*]:

$$D=\left[\begin{array}{ccc} x_{1} & y_{1} & z_{1}\\ \vdots & \vdots & \vdots \\ x_{N} & y_{N} & z_{N} \end{array}\right]$$

The proposed encoding technique requires three steps:

- Step 1: Normalize all ACC signals and scales to 255, converting as follows:
  (1) $$\bar{x}=\frac{x-min\left(X\right)}{max\left(X\right)-min\left(X\right)}\times 255\;\bar{y}=\frac{y-min\left(Y\right)}{max\left(Y\right)-min\left(Y\right)}\times 255\;\bar{z}=\frac{z-min\left(Z\right)}{max\left(Z\right)-min\left(Z\right)}\times 255$$
- Step 2: Convert the normalized ACC signal values into three integers that correspond to pixel values in the red, green, and blue channels of a color image, wherein each ACC signal value is treated as a pixel. For each sample of [*x y z*], three pixels are produced by our encoding technique:
  (2) $$R_{\bar{x}}=⎣\bar{x}⎦\;G_{\bar{x}}=⎣\left(\bar{x}-⎣\bar{x}⎦\right)\times 10^{2}⎦\;B_{\bar{x}}=⎣\left(\bar{x}\times 10^{2}-⎣\bar{x}\times 10^{2}⎦\right)\times 10^{2}⎦$$
- Step 3: Generate and write a color image I = [R G B] from a three-second ACC signal, including three color channels:
  (3) $$R=\left[\begin{array}{ccc} R_{{\bar{x}}_{1}} & R_{{\bar{y}}_{1}} & R_{{\bar{z}}_{1}}\\ \vdots & \vdots & \vdots \\ R_{{\bar{x}}_{N}} & R_{{\bar{y}}_{N}} & R_{{\bar{z}}_{N}} \end{array}\right]\;G=\left[\begin{array}{ccc} G_{{\bar{x}}_{1}} & G_{{\bar{y}}_{1}} & G_{{\bar{z}}_{1}}\\ \vdots & \vdots & \vdots \\ G_{{\bar{x}}_{N}} & G_{{\bar{y}}_{N}} & G_{{\bar{z}}_{N}} \end{array}\right]\;B=\left[\begin{array}{ccc} B_{{\bar{x}}_{1}} & B_{{\bar{y}}_{1}} & B_{{\bar{z}}_{1}}\\ \vdots & \vdots & \vdots \\ B_{{\bar{x}}_{N}} & B_{{\bar{y}}_{N}} & B_{{\bar{z}}_{N}} \end{array}\right]$$

The Algorithm 1 shows the process of ACC signal encoding:

**Algorithm 1.** ACC signal encoding

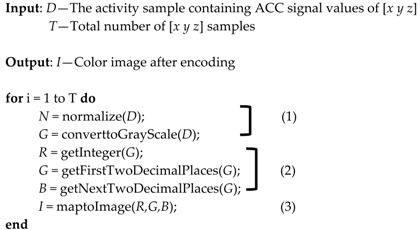



**Figure 2:**
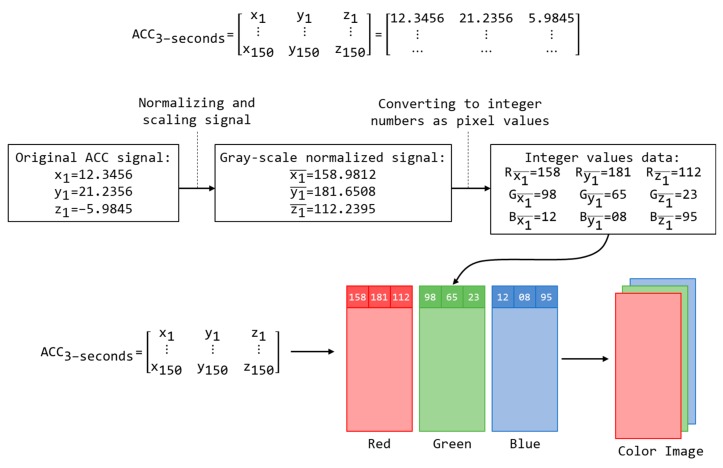
An example of encoding an ACC signal into an image.

Figure 2 shows an example of the ACC signal encoding procedure. Suppose a three-second set of ACC signals is input in which the first *x*, *y*, and *z* values are $x_{1}$ = 12.3456, $y_{1}$ = 21.2356, and $z_{1}$ = 5.9845. Those data are normalized first and then multiplied by 255 for gray-scale conversion, which changes the values to $\bar{x_{1}}$ = 158.9812, $\bar{y_{1}}$ = 181.6508, and $\bar{z_{1}}$ = 112.2935. Then, each axis value is mapped onto three integer values for the three corresponding color channels. For example, $\bar{x_{1}}$ will be mapped as $R_{{\bar{x}}_{1}}$ = 158, $G_{{\bar{x}}_{1}}$ = 98, and $B_{{\bar{x}}_{1}}$ = 12, $\bar{y_{1}}$ into $R_{y_{1}}$ = 181, $G_{y_{1}}$ = 65, and $B_{y_{1}}$ = 08, and $\bar{z_{1}}$ into $R_{z_{1}}$ = 112, $G_{z_{1}}$ = 23, and $B_{z_{1}}$ = 95. Each of these values is treated as a pixel, and thus together they form a color image.

The output of our proposed signal encoder is a color image. The size of the output color image varies based on the number of samples N in an activity sample D, the number of devices (smartphones and smartwatches), and number of sensors used for activity recognition. For example, an activity sample observed for three seconds using one smartphone and one smartwatch only collecting ACC signals has 150 [*x y z*] samples at a sampling rate of 50 Hz/s. The output color image is thus produced with a resolution of 150 × 6, where 150 is the number of samples and 6 is the *x*, *y*, and *z* components from the smartphone and smartwatch. The sampling rate parameter of all devices at the collection time must be the same for synchronization.

### 3.2. CNN-Based Human Activity Learning

**Figure 3:**
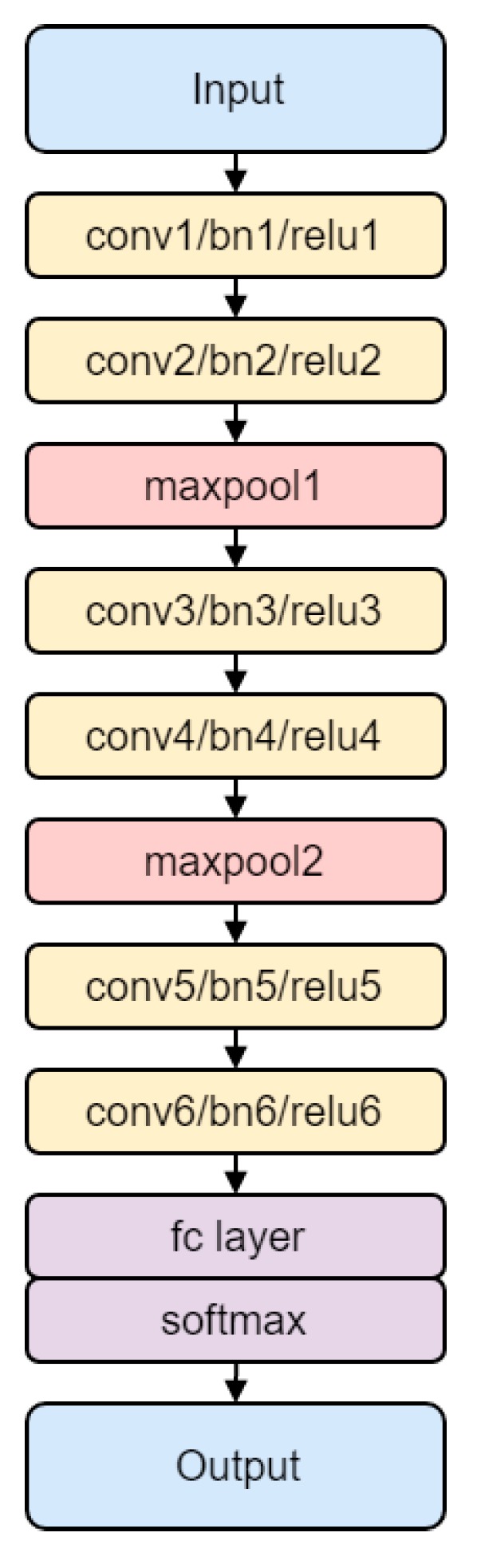
The architecture of UCNet6 for image-based activity recognition.

In this section, we contribute a CNN model for the deep learning HAR classification task after the input of an image. Each activity sample D is represented as an activity image I after applying our proposed encoding technique. In our method, the inertial sensor signal based HAR task becomes an activity image classification task using the deep learning technique. Therefore, the CNN model presented in this paper is generally designed to be suitable for image recognition. In particular, our CNN model, namely UCNet6, includes six convolutional layers in addition to the batch normalization layers, rectified linear unit (ReLU) layers, max pooling layers, fully connected layer, and softmax layer. The architecture of our CNN model is shown in Figure 3 and summarized in detail in Table 1.

In UCNet6, the six 2-D convolutional layers have a square filter size of 3, stride (or step size for traversing input) size of 1, and padding size of 1. After each convolutional layer, a batch normalization layer normalizes each input channel across a mini-batch, and a ReLU layer has as an activation function. There are three blocks of convolutional operation, and each block includes two modules of {2Dconvolutional, batch-normalization, and ReLU}. The two modules differ in the number of filters defined inside a convolutional layer: the first module has 64 filters, and the second module has 128 filters. Between the convolutional blocks is a max pooling layer to perform down-sampling by dividing the input into rectangular pooling regions. All the max pooling layers in our network are configured with a square pooling region size of 2, stride size of 2, and padding size of 0.

At the end of the network, we summarize with a fully connected layer and softmax function layer to classify an object with probabilistic values between 0 and 1. The fully connected layer for the classification task is defined with an output size equal to the number of classes, i.e., the number of activities in this research. At the bottom of the network, the classification layer holds the name of the loss function, particularly the cross entropy function that is used for training the network for multi-class classification. A convolutional network thus has two parts: feature learning (convolutional layer, batch normalization layer, ReLU layer, and pooling layer) and classification (fully connected layer and softmax layer).

The input layer is defined by the input image size, i.e., the resolution of the color image, where the image resolution depends on the activity observation duration (seconds), the sampling rate parameter (Hz or samples/second), and the number of devices. As explained in the previous subsection, the resolution of the output images created by the encoder is 150 × 6; therefore, the input size of the image input layer is set to 150 × 6.

## 4. Experiments and Discussion

This section benchmarks our proposed activity recognition method on our own collected dataset, UC-HAR, and three public datasets commonly used in this research field, MobiAct [36], DaLiAc [37], and UCI-HAR [38]. The proposed method is also compared with other state-of-the-art approaches in terms of recognition accuracy and processing speed.

### 4.1. Dataset

We used the following datasets:

- MobiAct: This dataset is from a single smartphone (Samsung Galaxy S3) positioned in the trouser pocket to collect data from an accelerometer, gyroscope, and orientation sensor at a 100 Hz sampling rate without considering orientation. Fifty four (54) subjects participated in data collection: 42 men and 15 women from 20 to 47 years old, of 160–189 cm height and 50–120 kg weight. Nine kinds of daily activities were performed: standing, walking, jogging, jumping, stairs up, stairs down, sit chair, car step in, and car step out.
- DaLiAc: This dataset is from four shimmer sensors attached to the left ankle, right hip, chest, and right wrist of participants to collect accelerometer and gyroscope data at a 200 Hz sampling rate. Data collection used 19 subjects—eight female and 11 male—26 ± 8 years old; their height was 177 ± 11 cm, and their weight was 75.2 ± 14.2 kg. Thirteen daily activities were performed: postures (sitting, lying, standing), household activities (washing dishes, vacuuming, sweeping), walking (normal walking, running, climbing stairs) and sports (bicycling at two speeds, rope jumping).
- UCI-HAR: This dataset is from a single smartphone (Samsung Galaxy S2) positioned on the user’s waist to collect accelerometer and gyroscope data at a 50 Hz sampling rate. Thirty subjects aged 19 to 48 years participated in data collection for six daily activities: walking, walking upstairs, walking downstairs, sitting, standing, and lying.
- UC-HAR: This dataset is from a smartphone (Samsung Galaxy S5) and a smartwatch (LG G Watch R) to collect accelerometer data at a 100 Hz sampling rate. The smartphone was positioned in the trousers pocket, and the smartwatch was positioned on the right wrist because all 28 male subjects who participated in the data collection were right handed; the subjects ranged in age from 20 to 30, in height from 163 cm to 185 cm, and in weight from 58 kg to 92 kg. None of the subjects had any kind of physical or mental disorder. Eight daily activities were collected: eating, lying, running, sitting, standing, stretching, sweeping, and walking. Each subject performed each activity for one minute. Thus, eight minutes of activity data were collected from each person.

Table 2 shows the activity list of all of the datasets and the abbreviations used in the confusion matrix of the experimental results.

### 4.2. Experimental Setup

We evaluated our proposed method with all of the datasets using fixed-width sliding windows of 3 s and an overlapping size of 1 s as the default configuration. As explained in the previous section, the resolution of the output image generated by the Iss2Image technique depends on the window size, the sampling rate, the number of devices, and the number of sensors. For example, with the UCI-HAR dataset, the image resolution is 150 × 6, where 150 represents the number of samples collected in 3 s at a sampling rate of 50 Hz, and 6 represents 3-axial linear accelerations and 3-axial angular velocities from the accelerometer and gyroscope. Based on the resolution of the input image, the size of the input layer in UCNet6 is also modified adaptively for each particular dataset. Additionally, we trained the convolutional network in 45 epochs using the stochastic gradient descent with momentum optimizer, a mini-batch size of 128, and initial learning rate of 0.1 with a 10-times downgrade after 15 epochs. We conducted the three following experiments using Matlab 2018b on a PC with a core i5 4.5 GHz CPU (Intel, Santa Clara, CA, USA), 16 Gb of memory, and a single GTX 1080ti GPU (Nvidia, Santa Clara, CA, USA):

- The first experiment evaluated the proposed method on human activity recognition using the various datasets.
- The second experiment compared our Iss2Image encoding technique with other transformation approaches that convert inertial sensor data to image-type data.
- The third experiment compared the performance of our UCNet6 model with other pre-trained models
- The last experiment compared the performance of our Iss2Image method and UCNet6 with existing methods in terms of recognition accuracy and processing speed.
- Image creation time among different sensor signal transformation methods inference time among different CNN models are also benchmarked.

### 4.3. Experimental Results and Discussion

**Figure 4:**
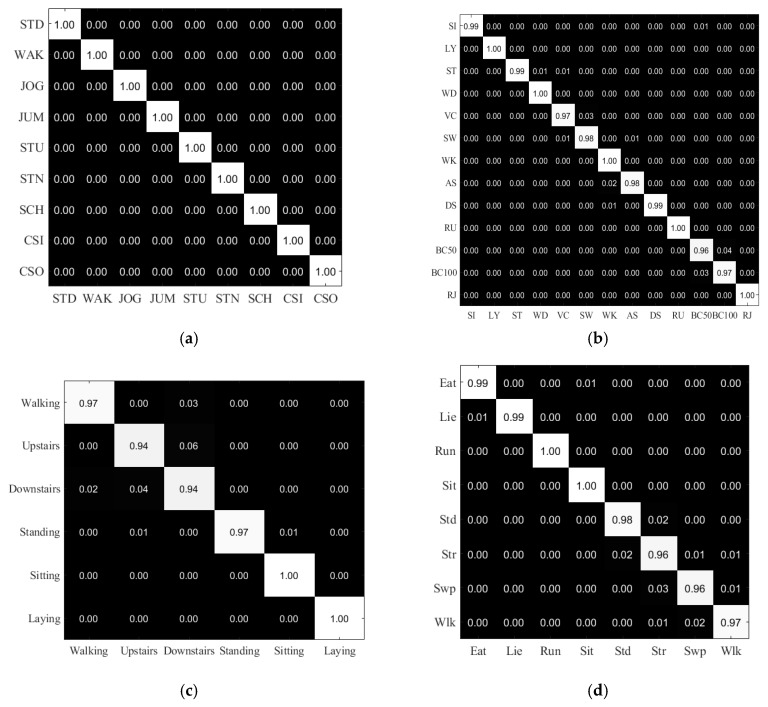
The confusion matrices of the recognition results of our proposed Iss2Image and UCNet6 method on the different datasets: (**a**) MobiAct, (**b**) DaLiAc, (**c**) UCI-HAR, and (**d**) UC-HAR.

In the first experiment, we evaluated our proposed method on the MobiAct, DaLiAc, UCI-HAR, and UC-HAR datasets. The recognition results are presented as confusion matrices in Figure 4 and summarized as average recognition accuracy in Table 3. With the MobiAct dataset, our method yields 100% recognition accuracy on the test set. The three remaining datasets are more challenging, but the overall accuracy is still impressive, with 98.9%, 97.11%, and 98.02% accuracy on the DaLiAc, UCI-HAR, and UC-HAR datasets, respectively. With the DaLiAc dataset, our method misrecognized some activities in the same group, for example, vacuuming with sweeping in HOUSE and bicycling 50 W with bicycling 100 W, because of the similarity of those activities in a realistic environment and the inhomogeneity of the actors.

The activities in the REST (sitting, lying, and standing) and WALK (walking, running, and ascending/descending stairs) groups are proficiently recognized with very high accuracy. In the UCI-HAR dataset, our method was confused between walking and downstairs and upstairs and downstairs, but sitting and lying were precisely recognized. This result is explained by the position of the smartphone during data collection for that dataset; all volunteers wore the smartphone on the waist. In our dataset, standing and stretching were confused because the time between the two consecutive stretching actions was considered as a standing activity. Furthermore, sweeping was sometimes detected as stretching because of the position of the smartwatch.

**Figure 5:**
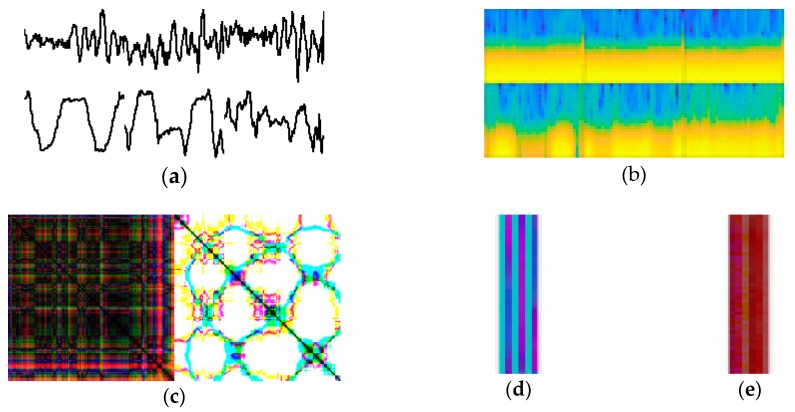
An illustration of activity images generated by (**a**) raw signal plot, (**b**) spectrogram, (**c**) recurrence plot, (**d**) multichannel, and (**e**) Iss2Image.

In the second experiment, we compared our proposed encoding technique with four other approaches that transform inertial sensor signal into an image for activity representation: the first one [39], called the raw signal plot method, transforms the acceleration signal directly into a time series image and represents it as a gray-scale image; the second one [40], called the spectrogram method, plots a spectrogram of an inertial signal after computing squared Short-Time Fourier Transform for input into a deep neural network; the third one [41], called recurrence plot method, a distance matrices that capture temporal patterns in the signal, represented in image with texture patterns; the last one [42], called the multichannel method, encodes the acceleration signal (including *X*, *Y*, and *Z*) into the corresponding red, green, and blue channels of a color image by normalizing, scaling, and rounding a real value into an integer for pixel representation. Some example activity images generated by the transformation methods are presented in Figure 5.

In this experiment, we evaluated and compared the accuracy of HAR using our deep network with the input images generated from each of the different transformation techniques on all four datasets. All of the benchmarked techniques are realized by our own implementation. In the comparison result reported in Table 4, Iss2Image is replaced by the other transformation methods without modifying our deep neural network. As shown in Table 4, our proposed encoding technique outperformed the other transformation methods for most of the benchmarked datasets: by 0.97% on MobiAct, 6.53% on DaLiAc, 4.87% on UCI-HAR, and 3.72% on UC-HAR on average. Compared with the raw signal plot, spectrogram and recurrence plot approaches, Iss2Image is much more powerful, 4.45%, 4.3% and 6.59% more accurate, respectively, on average across all datasets. Similar to Iss2Image, the multichannel approach encodes sensor signal sample of [*x y z*] to a pixel with three values for the red, green, and blue channels; however, it encodes only the integer of the real number instead of the integer plus four more decimal places used in Iss2Image. Thus, the precision of the multichannel approach is less than in our proposed technique, which lowers its accuracy by approximately 0.36% on average across all datasets. Clearly, plotting a raw signal, spectrogram or recurrence plot are not efficient solutions for representing activity signals in images because of distortion in the original information during the conversion and the complexity of the operation.

In the third experiment, we have compared the accuracy of our network (trained from scratch and pre-trained on CIFAR-10 [43]) with three other pre-trained CNNs, which are Resnet18 [44], Alexnet [45] and GoogleNet [46]. Note that the depth of our network is small, having six layers. Resnet18, containing 18 layers, introduces a deep residual learning framework. Instead of hoping each few stacked layers directly fit a desired underlying mapping, it explicitly let these layers fit a residual mapping to solve degradation problem. Alexnet, containing eight layers, adopted ReLUs, local response normalization and overlapping pooling to improve the performance and reduce training time. GoogleNet, containing 22 layers, is based on NIN (Network In Network) [47], adding Inception architecture which includes 1 × 1, 3 × 3 and 5 × 5 convolution for efficiency and then 1 × 1 convolution for dimension reduction to reduce the computation. All of these three networks classifies images into 1000 objects categories, based on the ImageNet database having 1.2 million images. To pre-train UCNet6, we have used CIFAR-10 dataset, having 50,000 training images and 10,000 testing images with 10 classes.

From the experimental results, the highest accuracy on different signal transformation methods is achieved by using ResNet18 and GoogleNet. Recurrence plot and Iss2Image method showed the highest accuracy on ResNet18 while raw signal plot, spectrogram and multichannel method showed highest results on GoogleNet. Raw signal plot has the lowest accuracy on all five networks, which indicates such approach has lowest efficiency on signal transformation. This can be inferred that having too much blank area on the image, convolves meaningless information. Spectrogram and recurrence plot showed average accuracy between raw signal plot and multichannel-Iss2Image group. On all of the networks, Multichannel and Iss2Image showed high accuracy over 98%, meaning that images made from these two methods are concise. UCNet6 trained from scratch shows the lowest performance on the overall average, especially on the high-resolution images, but shows comparable result on multichannel and Iss2Image. Pre-trained UCNet6 overcomes this weakness, showing comparable results on other three signal transformation methods, resulting average accuracy of 96.41%. Although this is still lower than other public pre-trained networks, it has the difference under 1%, ResNet18 with 0.66%, GoogleNet with 0.84% and Alexnet with 0.27%. Because the purpose of UCNet6 is for fast and lightweight, to be able to run on mobile platform in the future work, the performance is acceptable, and has the strength on training time and inference time which will be shown in the last part of this section. The three public pre-trained networks are pre-trained with very large data, having ability to classify images with richer feature maps. Pre-trained UCNet6 is trained with small-scale dataset, but possess the optimized parameters for learning, which can show comparable performance. Meanwhile, UCNet6 trained from scratch shows lowest performance but the gap is trivial. The recognition accuracy comparison of different transformation methods on different networks are shown in Table 5.

In the last experiment, we compared our proposed our method with state-of-the-art methods for HAR on the three public datasets, MobiAct, DaLiAc, and UCI-HAR, in terms of recognition accuracy. For a fair comparison, we strictly followed the benchmark setups (such as dataset partition and k-fold validation) indicated in the published research:

- MobiAct dataset: In the dataset paper [36], MobiAct was evaluated using a window size of 5 s with an overlapping ratio of 80%. The recognition accuracy was conducted using a conventional approach with three components: feature extraction, feature selection, and classification. In particular, the authors extracted and manually selected 64 features to train with the IBk and J48 classifiers. Both classifiers yield very high accuracy results of 99.88% and 99.30% on the MobiAct dataset, as shown in Table 6. However, this approach cannot precisely recognize mostly similar activities, such as stairs up and stairs down, due to the limitation of feature engineering. The method in [48] resampled the frequency to 20 Hz, segmented the data in 10 s window without overlapping, extracted features using Auto-Regressive model, and classified with SVM. But this approach also confused the similar activities, stairs up and stairs down, resulting 97.45% accuracy. Our Iss2Image-UCNet6 method consistently reports outstanding performance on MobiAct, with an average accuracy of 100%. In Iss2Image-UCNet6, many features produced inside the network by the convolutional layers, ReLU layers, and pooling layers are learned, producing better recognition accuracy than available with traditional classifiers, such as k-nearest neighbors and decision tree.
- DaLiAc dataset: Following the guidance in [37], we reimplemented our Iss2Image-UCNet6 method on DaLiAc with a 5-s window and 50% overlapping ratio. The comparison results are reported in Table 7, using the results for the other methods presented in the dataset paper. The authors extracted 152 features for each sliding window, including time and frequency domain features, and a hierarchical classification system including AdaBoost, a classification and regression tree, k-nearest neighbor (kNN), and SVM. The methods in [49,50] also extracted features from the acceleration signal in both the time and frequency domains; however, they each use a single classifier, decision tree and kNN, respectively. The paper in [51] divided the subjects of DaLiAc dataset into three subsets for training, validating and testing. 10 steps of feature extraction was conducted from original and magnitude time series data, and select features by discarding unimportant features and applying diversified forward-backward feature selection method. Comparing with six different classifiers, SVM showed the highest accuracy of 93%. Following the experiment configuration in [37], we evaluated the Iss2Image-UCNet6 method with a leave-one-subject-out procedure. In this comparison, our method outperformed the existing approaches with an impressive improvement in accuracy. Compared with traditional classification techniques, a deep neural network is much more powerful in classifying a large dataset. In addition, transforming the sensor signal into an image without much data distortion, as Iss2Image does, is important for achieving high recognition accuracy.
- UCI-HAR dataset: For a fair comparison, we benchmarked our proposed method with a sliding window size of 2.56 s and 50% overlap. The comparison results are reported in Table 8. In [52], the authors extracted 17 features measured in the time and frequency domains for both accelerometer and gyroscope data and classified the activities using a MultiClass SVM (MC-SVM). Additionally, they developed another lightweight version with fixed-point arithmetic for energy efficiency. The accuracy of the standard version and lightweight version of the MC-SVM is acceptable, approximately 89.30% and 89.00%. The authors of [19] proposed a HAR system using deep learning neural networks, entering the accelerometer and gyroscope sensor data after some preprocessing steps. To improve performance, those convolutional networks were combined with an MLP. This combination strategy produced 94.79% recognition accuracy on average. Another strategy described in [19] combines the features extracted from the convolutional layers with the features extracted by FFT. That strategy improves on the first strategy, with an average accuracy of 95.75%. The authors of [53] proposed a hierarchical classification named GCHAR which has two stage classification. The first stage is group based classification, dividing similar activities into a specific activity group. The second stage is context awareness based, correcting the activity to be included in the proper group from stage one. Comparing with six other traditional classifiers, GCHAR showed the highest accuracy of 94.16%. With our Iss2Image-UCNet6 method, the accuracy suffered from decreasing the window size from 3 s to 2.56 s, so in this experiment, Iss2Image-UCNet6 achieved an average accuracy of only 96.84%, but that is still better than the other methods. Our method is much better than the MC-SVM, whereas the accuracy improvement over Convnet is not significant. These results show the power of deep CNNs in classification tasks.

Finally, we benchmarked the sensor signal transformation time, training time and inference time for different networks using our collected dataset.

From the perspective of signal transformation time from inertial sensor signal to image, we have set the time for 10 s and count how many images were created for fair comparison. The raw signal plot and spectrogram method created only few images, less than 10 images. The recurrence plot created medium number of images about 700 images, and the multichannel method and our proposed method created the most with similar number of images, 2838 images and 2772 images, respectively.

Plotting *x*, *y*, and *z* signals into a single component image and combining all of the images to a unified image, raw signal plot method spends much time for signal transformation. Similarly, spectrogram method also spends much time for plotting spectrogram and writing it to an image. Meanwhile, recurrence plot captures temporal patterns in the signal first and then plot the overall texture pattern into image, shortening time than raw signal plot and spectrogram.

Compared with the above mentioned three methods, multichannel and our proposed approach takes less time for converting raw inertial signal to image due to directly encoding only raw data to pixel value. However, the computational cost of our method is more expensive than multichannel method because we have to encode not only the integer part but also the floating part. The comparison result of signal transformation time is shown in Table 9.

From the perspective of training time, we have compared ResNet18, GoogleNet, AlexNet, and trained from scratch and pre-trained UCNet6 with five different signal transformation methods. A total of 38,279 activity samples are segmented with a sliding window size of three seconds and an overlapping width of one second. We have made different sizes of images on different methods that the networks have different size of input image; 244 × 244 for ResNet18 and GoogleNet, 227 × 277 for AlexNet. The input size for UCNet6 networks are not fixed, having flexibility to be changed. Raw signal plot, spectrogram and recurrence plot have higher resolution while multichannel and Iss2Image has low resolution.

Training time for public pre-trained networks costed similar times; average time about 62 min for ResNet18, 57 min for GoogleNet, and 74 min for AlexNet. On each network, training time on different signal transformation methods also did not show big differences; the time gap between highest and lowest time is 4 min for ResNet18, 6 min for GoogleNet, and 7 min for AlexNet. Iss2Image was fastest in GoogleNet among other signal transformation methods, but not from the others. But Iss2Image is still competitive on other networks that it was third fast on ResNet18 and second fast on AlexNet.

The UCNet6 trained from scratch took more than 5 h for training for raw signal plot, spectrogram and recurrence plot. Without utilizing pre-trained parameters, it took long time on high resolution images for reaching desired training performance. On the contrary, training time for multichannel and Iss2Image took very short, 7 min and 9 min respectively, showing that the way of initializing parameters (from random generation or from pre-trained model) does not impact on training speed of low resolution image. The pre-trained UCNet6 showed the highest performance, having average speed of 9 min. By utilizing transfer learning, it now has the ability to well handle the high resolution images. The comparison result of training times on different networks is shown in Table 10.

From the perspective of inference time, we have compared ResNet18, GoogleNet, AlexNet and our network with five different signal transformation methods. A total of 1000 activity samples are segmented with a sliding window size of three seconds and an overlapping width of one second. We have made different sizes of images on different methods that the networks have different size of input image; 244 × 244 for ResNet18 and GoogleNet, 227 × 277 for AlexNet, and 150 × 6 for UCNet6. The inference time is shown by calculating the average for 10 times execution.

As shown in the result on Table 11, all of the five different methods showed fastest inference time on UCNet6 than other pre-trained networks where the average speed is less than one second. This is because that having less layers with less complex layers, an input image will pass the network end-to-end with less computation. Among three pre-trained networks, GoogleNet has the most number of layers but faster than ResNet18. This is because ResNet18 contains more complex layers such as batch normalization and addition layers, resulting in more computation time. In the case of AlexNet, it has relatively small number of layers than other two networks which is able to compute fastest among three networks.

From the perspective of signal transformation methods, all three pre-trained networks showed the Iss2Image as second or third fastest method, compared with multichannel method, where the difference is negligible. The fastest was the raw signal plot, but this is not practical in real-time recognition environment that creating the image takes too much time. From UCNet6, because it is optimized for Iss2Image, the multichannel and Iss2Image showed the first and second fastest and faster than raw signal plot.

## 5. Conclusions

In this research, we have proposed an activity recognition method using a CNN. The Iss2Image encoder transforms the inertial sensor signals into an image form, and an image-based CNN model classifies the activity. The sensor signals are divided into three parts, the integer, the first two decimal places, and the following two decimal places, to minimize data distortion. In this way, we infer detailed correlations among successive sensor signal values in three different dimensions. We have also proposed UCNet6 which is a fast and lightweight CNN model. We compared the results of our proposed method with different datasets, different signal transformation methods, different CNNs, and different traditional methods, and benchmarked the speed of image creation time and inference time. The experimental results show that our proposed method outperforms existing methods using both our own dataset and public datasets. Our future work will incorporate the improvements in accuracy reported here into different datasets using different combinations of sensor signals, and also adopt this for real-time mobile based activity recognition.

## Figures and Tables

**Table 1:** The detailed CNN architecture of UCNet6.

Layer	No. of Filters	Size of Filer/Pooling	Size of Stride	Size of Padding
conv1	64	3	1	1
conv2	128	3	1	1
maxpool1	-	2	2	0
conv3	64	3	1	1
conv4	128	3	1	1
maxpool2	-	2	2	0
conv5	64	3	1	1
conv6	128	3	1	1

**Table 2:** Activity list for all the datasets and their abbreviations.

MobiAct	DaLiAc	UCI-HAR	UC-HAR
Standing (STD)	Sitting (SI)	Walking	Eating (Eat)
Walking (WAK)	Lying (LY)	Upstairs	Lying (Lie)
Jogging (JOG)	Standing (ST)	Downstairs	Running (Run)
Jumping (JUM)	Washing dishes (WD)	Standing	Sitting (Sit)
Stairs up (STU)	Vacuuming (VC)	Sitting	Standing (Std)
Stairs down (STN)	Sweeping (SW)	Lying	Stretching (Str)
Stand to sit (SCH)	Walking (WK)		Sweeping (Swp)
Car step in (CSI)	Ascending stairs (AS)		Walking (Wlk)
Car step out (CSO)	Descending stairs (DS)		
	Running (RU)		
	Bicycling on ergometer 50 W (BC50)		
	Bicycling on ergometer 100 W (BC100)		
	Rope jumping (RJ)		

**Table 3:** Average recognition accuracy of our proposed method on the different datasets.

Dataset	Accuracy (%)
MobiAct	100.00
DaLiAc	98.90
UCI-HAR	97.11
UC-HAR	98.16

**Table 4:** Average recognition accuracy (%) comparison of the different transformation methods with the different datasets.

Method	MobiAct	DaLiAc	UCI-HAR	UC-HAR
Raw signal plot [39]	98.22	92.06	92.86	93.08
Spectrogram [40]	98.02	94.54	91.02	93.40
Recurrence plot [41]	100.00	84.75	88.47	93.15
Multichannel [42]	99.88	98.12	96.60	98.14
Iss2Image	100.00	98.90	97.11	98.16

**Table 5:** Recognition accuracy (%) comparison of different transformation methods on different networks.

Method	ResNet18	GoogleNet	AlexNet	UCNet6 (from Scratch)	UCNet6 (Pre-Trained)
Raw signal plot	93.82	94.55	94.15	93.08	94.24
Spectrogram	95.19	96.87	96.09	93.40	95.46
Recurrence plot	98.10	96.38	96.15	91.15	94.37
Multichannel	98.55	99.02	98.07	98.14	98.71
Iss2Image	99.70	99.46	98.97	98.16	99.27
Average	97.07	97.25	96.68	95.78	96.41

**Table 6:** Average accuracy comparison between Iss2Image-UCNet6, and existing methods on the MobiAct dataset.

Method	Accuracy (%)
IBk [36]	99.88
J48 [36]	99.30
SVM [48]	97.45
Iss2Image-UCNet6	100.00

**Table 7:** Average accuracy comparison between Iss2Image-UCNet6 and existing methods on the DaLiAc dataset.

Method	Accuracy (%)
Hierarchical classifier [37]	89.60
Decision tree [49]	80.00
kNN [50]	68.70
SVM [51]	93.00
Iss2Image-UCNet6	96.40

**Table 8:** Average accuracy comparison between Iss2Image-UCNet6 and existing methods on the UCI-HAR dataset.

Method	Accuracy (%)
MC-SVM [52]	89.30
Convnet + MLP [19]	94.79
tFFT + Convnet [19]	95.75
GCHAR [53]	94.16
Iss2Image-UCNet6	96.84

**Table 9:** Comparison of image creation time on different signal transformation methods.

Method	Created Images for 10 s
Raw signal plot	7 images
Spectrogram	4 images
Recurrence plot	699 images
Multichannel	2838 images
Iss2Image	2772 images

**Table 10:** Comparison of training times (minutes) on different networks.

Method	ResNet18	GoogleNet	AlexNet	UCNet6 (from Scratch)	UCNet6 (Pre-Trained)
Raw signal plot	60	57	73	309	9
Spectrogram	63	58	71	330	8
Recurrence plot	64	60	77	318	14
Multichannel	61	58	78	7	6
Iss2Image	62	54	72	9	7
Average	62	57	74	195	9

**Table 11:** Comparison of inference time (seconds) on different networks with 1000 samples.

Method	ResNet18	GoogleNet	AlexNet	UCNet6
Raw signal plot	1.89	1.77	1.18	0.61
Spectrogram	2.06	1.90	1.33	0.87
Recurrence plot	2.12	1.93	1.33	0.88
Multichannel	1.96	1.86	1.30	0.17
Iss2Image	1.99	1.87	1.27	0.29
Average	2.01	1.87	1.29	0.56
